# The Outcome of Multicystic Dysplastic Kidney Disease Patients at King Abdulaziz Medical City in Riyadh

**DOI:** 10.7759/cureus.37994

**Published:** 2023-04-22

**Authors:** Abdulrahman Alamir, Soud A Al Rasheed, Abdullah T Al Qahtani, Mohammad S Almosa, Nawaf D Aljehani, Eid D Alanazi, Khalid A Almutairi

**Affiliations:** 1 Department of Pediatric Nephrology, King Abdullah Specialized Children's Hospital, Riyadh, SAU; 2 College of Medicine, King Saud Bin Abdulaziz University for Health Sciences, Riyadh, SAU

**Keywords:** genitourinary abnormalities, vesicoureteric reflux, multicystic dysplastic kidney, mcdk, outcome of mcdk, multicytic

## Abstract

Background

Multicystic dysplastic kidney (MCDK) is a type of kidney dysplasia consisting of many irregular, various-sized cysts divided by dysplastic renal tissue, which negatively impacts kidney function. MCDK is one of the most common renal congenital disorders seen in antenatal ultrasounds. The typical prognosis of MCDK is complete or partial involution that starts antenatally and continues postnatally. The aim of the study was to shed light on the overall outcome of patients with MCDK.

Methods

We retrospectively collected data on MCDK patients from 2016 until 2022 at King Abdulaziz Medical City, Ministry of National Guard Health Affairs in Saudi Arabia, Riyadh. The data included the recording of epidemiological data, radiological and laboratory reports, and the presence of urological or non-urologically associated anomalies.

Results

A total of 57 patients with MCDK were reviewed. Seven of them were excluded due to the diagnosis of bilateral MCDK, which was incompatible with life. Of the remaining 50 patients, the right kidney was affected in 52% of them. Most patients were diagnosed antenatally (98%). The mean duration of follow-up for the study was 48 months. Vesicoureteral reflux (VUR) was detected in 22% of the total sample. Overall, 90% of the patients underwent kidney involution. A small percentage had genitourinary anomalies (20%), while a larger percentage (48%) had extrarenal abnormalities.

Conclusion

Multicystic dysplastic kidney disease is relatively common in children. The prognosis is affected by the presence of genitourinary and non-genitourinary anomalies. Patients have an overall good prognosis with conservative management. Antenatal screening, diagnosis, and long-term nephrological follow-up are essential for the optimal management of patients.

## Introduction

Renal cystic diseases are multiple heterogeneous conditions characterized by the existence of multiple cysts that develop in the kidneys. These conditions are one of the primary causes of chronic kidney disease (CKD). Renal cystic diseases are categorized in numerous different ways. However, it is clinically beneficial to classify them as genetic disorders, such as autosomal dominant polycystic kidney disease and autosomal recessive polycystic kidney disease, and non-genetic disorders, such as multicystic dysplastic kidney (MCDK), as this method is helpful in assessing and managing the patients [1].

Multicystic dysplastic kidney (MCDK) is a type of kidney dysplasia consisting of many irregular, variously sized, non-communicating cysts divided by dysplastic renal tissue, which has harmful effects on kidney function. Therefore, kidney function relies on the normal contralateral kidney. MCDK is one of the most common renal congenital disorders seen in antenatal ultrasound, with an incidence of one out of every 4300-4500 live deliveries [2, 3].

The majority of MCDK cases are unilateral and isolated. The typical prognosis of MCDK is complete or partial involution that starts antenatally and continues postnatally [4]. In a meta-analysis of 67 studies with around 3500 patients, a small left-sided predominance (53%) was found [5]. Around 60%-80% of MCDK cases are diagnosed antenatally. However, approximately 20% of MCDK cases are detected postnatally. Patients may present with symptoms such as a urinary tract infection or an abdominal mass, or it may be an incidental finding [5].

Previous studies have shown that most MCDK kidneys eventually involute within the first five years of life. [6,7,8] A conservative approach to children with MCDK has subsequently been recommended. Nevertheless, others have proposed surgical excision to minimize the risk of developing hypertension, mass effect, and malignant change, as well as reduce the cost of serial ultrasound examinations [9,10,11].

The nephrological follow-up of MCDK patients is essential. In our study, we retrospectively reviewed and analyzed the records of children with MCDK to assess the outcome, clinical course, parameters, and related renal and urological anomalies.

## Materials and methods

We retrospectively collected data on multicystic dysplastic kidney disease (MCDK) patients at King Abdulaziz Medical City, Ministry of National Guard Health Affairs in Saudi Arabia, Riyadh. The population of the study included pediatric patients with multicystic dysplastic kidney disease diagnosed from 2016 until 2022. Patients’ data were obtained by reviewing their electronic medical files. We included all patients diagnosed with MCDK, both antenatally and postnatally. All cases of MCDK that were incompatible with life were excluded, as were patients who were followed up for less than a year.

The collection of data included the recording of demographic data, history of consanguinity, blood pressure, electrolytes, urine analysis, ultrasound, dimercaptosuccinic acid (DMSA) radionuclide scan, micturating cystogram (MCUG), creatinine, and blood urea nitrogen (BUN) at presentation and on the last follow-up. Additionally, the presence of a urinary tract infection history, kidney involution, urological abnormalities, and non-urological anomalies was noted. We calculated the estimated glomerular filtration rate (e-GFR) using the Schwartz formula. Hypertension was defined as blood pressure (BP) above the 95 percentile for age, height, and sex.

Our aim is to evaluate the outcome of children with antenatally or postnatally detected MCDK by documenting complications, the size of the affected kidney, and renal function. Furthermore, documentation of compensatory changes in the contralateral kidney was taken into consideration, which involved changes in size, function, and the presence of vesicoureteral reflux (VUR) or any other abnormalities.

This research is integrated with human individuals, so ethical issues were considered. We maintained complete confidentiality during the collection and analysis of the patient’s data. Moreover, to avoid harm and ensure privacy, patients’ medical information was kept anonymous. No medical record numbers or names were taken. Informed consent was not needed as the study was conducted through a chart review. Ethical approval was obtained for this study by the Institutional Review Board of King Abdullah International Medical Research Center, Ministry of National Guard Health Affairs, Riyadh, Kingdom of Saudi Arabia (IRB/2435/22).

Statistical analysis

The data were recorded using Microsoft Excel and presented in the form of tables. R Studio software (version 2022.02.3, The R Foundation for Statistical Computing, Boston, USA) was used for data analysis. The normality of the data distribution was checked using the Kolmogorov-Smirnov test. Continuous normally distributed variables were presented as mean (± SD) and compared using an independent t-test. Continuous non-normally distributed variables were presented as medians (25th percentile, 74th percentile) and compared using the Wilcoxon test.

Categorical variables were presented as frequencies (%) and compared using Pearson’s Chi-squared test or Fisher’s exact test. Univariate logistic regression was done for all variables, and the odds ratio (OR, 95% confidence intervals) was reported.

A multivariate logistic regression analysis was used to correlate various factors with the risk of readmission. Factors reaching or approaching statistical significance (p ≤ 0.1) in the univariate analysis were included in the multivariate model. For all analyses, p < 0.05 was considered statistically significant.

## Results

A total of 57 patients with MCDK were reviewed. Seven of them were antenatally diagnosed with bilateral MCDK that was incompatible with life and died due to hypoplastic lungs, thus being excluded from the study. Of the remaining 50 patients, the right kidney was affected in 26 (52%), and the left kidney in 24 (48%) patients. The majority of cases were diagnosed antenatally (49 children; 98%). All of the samples were from Saudi patients. The median gestational age was 38 weeks. The mean duration of follow-up for the study was 48 months. Table 1 presents the baseline characteristics of this cohort.

**Table 1:** Baseline characteristics and demographics of the sample MCDK: multicystic dysplastic kidney disease; IQR: interquartile range

Variable	Median (IQR)
Gestational age	38.00 (37.00, 39.00)
	Frequencies (Percentages)
Gender	
Male	31.00 (62%)
Female	19.00 (38%)
Consanguinity	3.00 (6%)
Family history of MCDK	3.00 (6%)
Family history of other renal or cystic diseases	3.00 (6%)

Out of 50 participants, VUR was detected in 11 patients (22%). Three of them didn’t have associated hydronephrosis. Two of the hydronephrosis-free patients had low-grade VUR reflux, which resolved spontaneously. Most of the VUR cases were in the contralateral kidney. Three VUR cases affected the dysplastic kidney, all of which were grade I. In the contralateral VUR, five out of seven cases were low-grade, while the remaining two patients had grade III and IV VUR. None of the patients with VUR in this study required surgery. Overall, 45 (90%) patients underwent kidney involution, while five patients (10%) had no kidney involution, which might be due to the short period of follow-up. All the patients had abnormal dimercaptosuccinic acid (DMSA) results for the affected kidney and normal results for the contralateral kidney. Table 2 summarizes MCDK outcomes in the studied population

**Table 2:** Clinical and radiological characteristics of the sample VUR: vesicoureteral reflux; SD: standard deviation

Variable	Mean (±SD)
Age of complete involution in months	21.38 (±14.47)
	Frequencies (Percentages)
Kidney involution	
Partial	34.00 (75.6%)
Complete	11.00 (24.4%)
Micturating cystourethrogram	
Normal	39.00 (78%)
Reflux into the contralateral kidney	7.00 (14%)
Reflux into the affected Kidney	3.00 (6%)
Bilateral	1.00 (2%)
VUR Associated with hydronephrosis	8.00 (72.7%)

Among the 50 patients included, only eight developed a urinary tract infection (UTI). Additionally, 17 patients were prescribed prophylactic antibiotics. Thirteen of them were given antibiotics due to a reflex detected by micturating cystourethrogram (MCUG), while the remaining four patients were on antibiotics due to one or more previous UTIs. The median glomerular filtration rate (GFR) was 83 (68.25, 90) mL/min/1.73 m2. The systolic and diastolic blood pressure readings were within the normal range in all the samples. Table 3 presents the laboratory results of the studied patients.

**Table 3:** Laboratory investigation and urological history of the sample BUN: blood urea nitrogen; UTI: urinary tract infection; (µmol/L): micromole per liter; (mmol/L): millimole per liter; IQR: interquartile range

Variable	Median value (IQR)
Creatinine at presentation (µmol/L)	52.00 (43.50, 67.50)
Creatinine at last visit (µmol/L)	43.00 (40.00, 48.00)
BUN at presentation (mmol/L)	2.80 (2.15, 3.80)
BUN at last visit (mmol/L)	3.75 (2.42, 4.80)
	Frequencies (Percentages)
UTI	8.00 (16%)
Prophylactic antibiotics	17.00 (34%)
Proteinuria	0.00 (0%)
Hematuria	0.00 (0%)

A small percentage of the studied sample had genitourinary anomalies (20%). Three (6%) patients had pelvi-ureteric junction obstruction, all of whom underwent pyeloplasty surgeries. Likewise, undescended testes were present in a total of three patients (6%). On the other hand, a larger percentage had extrarenal abnormalities (48%). Cardiac disease was found to be the most common extrarenal abnormality in a total of 12 patients (24%); half of the cases were septal defects; three cases were coarctation of the aorta; two were patent ductus arteriosus; and only one was tetralogy of fallout. Table 4 presents the associated anomalies in our cohort.

**Table 4:** Associated genitourinary and extrarenal anomalies in the sample

Variable	Frequencies (Percentages)
Genitourinary abnormalities	
Pelvi-ureteric junction obstruction	3.00 (6%)
Undescended testes	3.00 (6%)
Bladder diverticulum	2.00 (4%)
Kidney stones	1.00 (2%)
Small ureterocele with upper pole caliectasis	1.00 (2%)
Extrarenal abnormalities	
Cardiac	12.00 (24%)
Neurological	7.00 (14%)
Imperforated anus	3.00 (6%)
Adrenal hyperplasia	1.00 (2%)
Hirschsprung disease	1.00 (2%)

The size of the affected kidney decreased from a median of 4.90 cm at the initial assessment to a median of 2.70 cm at five years. In contrast, the size of the contralateral kidney increased from a median of 4.90 cm at the initial assessment to a median of 9.30 cm at five years. The corticomedullary differentiation of the affected kidney was preserved in 7.6% of the cases at the initial assessment, and the percentage decreased to 3.8% at two years and was 0% at five years. The corticomedullary differentiation of the contralateral kidney was preserved in 93.62% of the cases at the initial assessment, 97.87% at one year, and 100% at two and five years. The echogenicity of the affected kidney was normal in 23% of the cases at the initial assessment, 17.3% at one year, 9.6% at two years, and 0% at five years. The echogenicity of the contralateral kidney was normal in 95.74% of the cases at the initial assessment, 97.78% at one year, and 100% at two and five years. Table 5 presents the change in renal radiological characteristics over five years.

**Table 5:** Changes in the renal ultrasonographic characteristics over five years. cm: centimeters; IQR: interquartile range

Characteristic	Initial assessment	One year	Two years	Five years
	Median (IQR)			
Size of the affected kidney in cm	4.90 (3.22, 6.20)	4.20 (2.70, 5.47)	4.05 (2.10, 5.45)	2.70 (0.00, 4.98)
Size of the contralateral kidney in cm	4.90 (4.34, 5.15)	6.95 (6.40, 7.43)	7.70 (7.23, 8.30)	9.30 (8.80, 9.60)
	Percentages			
Preserved corticomedullary differentiation of the affected kidney	(7.6%)	(5.7%)	(3.8%)	(0%)
Preserved corticomedullary differentiation of the contralateral kidney	(93.62%)	(97.87%)	(100.00%)	(100.00%)
Normal echogenicity of the affected kidney	(23%)	(17.3%)	(9.6%)	(0%)
Normal echogenicity of the contralateral kidney	(95.74%)	(97.78%)	(100.00%)	(100%)

## Discussion

In our study, we assessed the outcomes of 50 MCDK patients. We observed a degree of kidney involution in the vast majority of the patients in a relatively short period of follow-up. Additionally, associated renal and extrarenal abnormalities were present in a considerable number of the patients. A previous local study reported that MCDK is the most common renal cystic disease at King Abdulaziz University Hospital [12]. Most of our patients were diagnosed antenatally, which is consistent with previous studies that indicate the importance of routine prenatal ultrasound [12]. Such a high percentage of MCDK detection reflects advancements in antenatal screening in King Abdulaziz Medical City.

Most of our patients were investigated consistently, with a mean follow-up of 48 months. However, discontinuity of follow-up was noted in some of the MCDK patients. This could be attributed to the reassuring prognosis of the disease. As one study suggests, urological follow-up is unnecessary in such patients [12]. During each follow-up visit, the physicians are advised to check blood pressure, perform an abdominal examination, and review laboratory tests including eGFR, creatinine, and urinalysis.

Contralateral kidney hypertrophy as a compensatory mechanism was reported in half of our sample, which is considered a low percentage when compared to a previous study where 77% of the patients developed compensatory hypertrophy [13]. Furthermore, other studies with a longer duration of follow-up illustrated higher percentages of hypertrophy, reaching 86% [14]. On the other hand, a decreased percentage rate of 43% was documented in previous literature; however, this percentage may be explained by the short period of follow-up [4]. In our cohort study, 90% of the patients demonstrated a degree of kidney involution. Out of the involution cases, we found that 24.4% were complete involutions. This is consistent with previous reports, where 25% of the patients developed complete involution after being followed up for a mean of 41 months [15]. The small percentages of complete involution in our study might be related to the short period of follow-up. The rate of complete involution increases with time; the complete involution rates were previously reported to be 9.8% at one year, 38.5% at five years, and 53.5% at ten years of age [16].

Hypertension does not usually develop in MCDK patients; previous articles have shown that among 1115 patients, only six had hypertension [4]. If hypertension develops, such a condition can be managed by nephrectomy [9]. However, hypertension may linger after the procedure [17]. All patients presented in our study had normal blood pressure throughout the span of follow-up. Apart from this, a variety of other genitourinary tract abnormalities and extrarenal anomalies were found in MCDK patients. Pelvi-ureteric junction obstruction was detected in 6% of the patients, which is comparable to a previous study that found it to be 5%. Furthermore, findings showed that 48% of the patients had coexisting extrarenal anomalies, in contrast to only 6.8% described in a previous local study [18].

The micturating cytogram (MCUG) was previously considered a standard diagnostic test performed postnatally in patients suspected of having MCDK. MCUG was performed in our study sample, showing that 22% of patients had vesicoureteral reflux. Some studies showed a similar incidence of vesicoureteral reflux (VUR), with percentages ranging from 4.5% to 28% of the patients [19,20,21]. Multiple studies have shown that the majority of VUR diagnosed on MCUG is low grade, most of which resolves spontaneously [22,23,24]. In addition to the MCUG, dimercaptosuccinic acid (DMSA) is also an essential imaging modality to diagnose a non-functioning kidney. In a previous study, DMSA was used in their samples, revealing abnormal renal function in all the affected kidneys [25]. Similarly, all patients in our study had abnormal DMSA results for the affected kidney.

Proteinuria was not reported in any of our patients. Patients had a normal electrolyte level with an overall reassuring outcome. Laboratory tests showed the median creatinine and blood urea nitrogen (BUN) values were 52 (43, 67.5) µmol/L and 2.8 (2.15, 3.8) mmol/L at presentation, respectively, and 43 (40, 48) µmol/L and 3.75 (2.42, 3.8) mmol/L at the last visit, respectively. A recent study conducted in 2019 documented that the maximum BUN (7.2 vs. 4.8, P = 0.041) and creatinine (110 vs. 69, P = 0.045) were significantly higher in infants with complex MCDK [26].

The main strength of our study is that it describes the outcome of MCDK in one of the largest tertiary care centers in Saudi Arabia. In addition, the presentation and outcomes of MCDK among our population are consistent with the local and international literature. In our study, we emphasize the importance of antenatal screening and the necessity of follow-up. Our study has a few limitations, including the observational nature of the study and being limited to a single center. Additional limitations include the relatively small sample size and the short period of follow-up.

## Conclusions

Multicystic dysplastic kidney disease is relatively common in children. The prognosis is affected by the presence of genitourinary and non-genitourinary anomalies, but patients have an overall good prognosis with conservative management. Antenatal screening and diagnosis are beneficial for optimal patient management. Patients should be followed up for long periods of time. In this study, the period of follow-up was longer among simple cases in view of the higher mortality among complex cases. Though MCUG is helpful in most cases, studies advocate against routine imaging due to its low clinical significance.
